# Epigenetic Regulation of Adipocyte Differentiation by a Rho Guanine Nucleotide Exchange Factor, WGEF

**DOI:** 10.1371/journal.pone.0005809

**Published:** 2009-06-05

**Authors:** Takuro Horii, Sumiyo Morita, Mika Kimura, Izuho Hatada

**Affiliations:** Laboratory of Genome Science, Biosignal Genome Resource Center, Institute for Molecular and Cellular Regulation, Gunma University, Maebashi, Japan; Deutsches Krebsforschungszentrum, Germany

## Abstract

Epigenetic regulation, including DNA methylation, plays an important role in several differentiation processes and possibly in adipocyte differentiation. To search for genes that show methylation change during adipogenesis, genome-wide DNA methylation analysis in insulin-induced adipogenesis of 3T3-L1 preadipocyte cells was performed using a method called microarray-based integrated analysis of methylation by isoschizomers (MIAMI). The MIAMI revealed that *Hpa* II sites of exon 1 in a Rho guanine nucleotide exchange factor 19 (ARHGEF19; WGEF) gene were demethylated during adipocyte differentiation of 3T3-L1 cells. Deletion of the region containing cytosine-guanine (CpG) sites that showed methylation change suppressed transcriptional activity in the reporter assay, indicating that this region regulates WGEF transcription. WGEF expression in 3T3-L1 cells was reduced during adipocyte differentiation, and high-fat diet-induced obese mice also showed lower expression of WGEF gene than control mice in white adipose tissue. Additionally, forced expression of WGEF in 3T3-L1 cells down-regulated the expression of adipogenic marker genes and inhibited the adipogenic program. This study clarified that adipogenesis was regulated by WGEF expression through DNA methylation change.

## Introduction

5-methylcytosine is the only covalent DNA modification known in vertebrates [1]. This epigenetic modification regulates gene expression and is essential for differentiation, embryonic development [2], genomic imprinting [3], and X-chromosome inactivation [4]. Recently, it was reported that methylation change of six CpG sites in a retrotransposon upstream of the transcription start site of the *Agouti* gene was responsible for obesity in *A^vy^/a* mutant mice [5], [6]. Accumulation of adipocytes usually results in obesity, and genes that regulate adipogenesis could be regulated by epigenetic DNA modifications; thus, it is very important to search for adipogenic genes that are regulatd by DNA methylation.

Previously, we developed a genome-wide DNA methylation analysis called MIAMI using a microarray [7]. With this method, we detected DNA methylation using the methylation-sensitive restriction enzyme *Hpa* II and its methylation-insensitive isoschizomer *Msp* I. *Hpa* II cleavage differences are usually related to methylation differences of two samples. This method has already been utilized for genome-wide profiling of lung cancer [7] and neural differentiation [8]. In this study, the MIAMI method was applied to genome-wide DNA methylation analysis for insulin-induced adipogenesis of 3T3-L1 preadipocyte cells, and we found a dramatic methylation change of Rho guanine nucleotide exchange factor 19 (ARHGEF19; WGEF) [9] during adipocyte differentiation.

WGEF is one of the members of Rho guanine nucleotide exchange factors (RhoGEFs). RhoGEFs stimulate the exchange of GDP for GTP to generate the activated form of GTPases, which is then capable of recognizing downstream targets, or effector proteins [10]. The Rho family of small GTPases, activated by RhoGEFs, has been shown to regulate a variety of cytoskeletal-dependent cell functions, such as cell morphology changes, formation of focal adhesions and stress fibers, platelet aggregation, cytokinesis, cell-cycle progression, and neurite outgrowth and guidance [11]. In addition, Rho family proteins are also involved in the differentiation of many cell types, including neurons, T lymphocytes, myocytes and keratinocytes [12]–[15]. Thus, RhoGEFs have important roles in many physiological and pathophysiological mechanisms via the activation of Rho GTPases. On the other hand, WGEF is expressed mainly in the intestine, liver, heart, and kidney, and it can mainly activate RhoA [9]. According to another research, RhoA inhibits adipogenesis by the regulation of cytoskeletal tension through the RhoA-Rho kinase (ROCK) signaling pathway [16]. These two reports are associated with the existence of adipogenic regulation through the WGEF-RhoA-ROCK signaling pathway; however, no direct evidences have been demonstrated.

Here, we show that epigenetic regulation, including DNA methylation in the WGEF gene, plays an important role in transcriptional activity, and we also prove that WGEF regulates adipocyte differentiation through the WGEF-RhoA-ROCK signaling pathway.

## Materials and Methods

### Cell culture

3T3-L1 cells were grown and maintained in DMEM containing 10% fetal bovine serum (Gibco). For adipocyte differentiation, confluent cells (day1) were treated with standard growth medium described above supplemented with 1.7 M insulin (Sigma), 0.5 µM dexamethasone (Sigma), and 0.8 mM isobutylmethyl xanthine (IBMX, Sigma) for 2 days. After 2 days (day3), this medium was replaced by medium supplemented with 1.7 M insulin only. Y-27632 (Wako Pure Chemical Industries) known as a ROCK inhibitor, was supplemented with differentiation culture medium as necessary.

### Mice

C57BL/6J mice were purchased from Charles River Japan. Six-week-old mice were housed in box cages, maintained on a 12-h light/12-h dark cycle and fed for 15 weeks either a normal diet (CE-2, CLEA Japan) or a high-fat diet (HFD32, CLEA Japan). All animal experiments were conducted according to the guidelines of the Animal Care and Experimentation Committee, Gunma University, Showa Campus, Japan.

### Methylation profiling by MIAMI

Transcriptional start sites for the genes were characterized on the basis of the National Center for Biotechnology Information (NCBI) annotation and/or Database of Transcriptional Start Sites (DBTSS). A DNA sequence around a transcriptional start site was extracted for each gene and subjected to megaBlast to select regions whose scores relative to the mouse genome were less than 30.0. In these regions all possible 60-mer probes in Hpa II fragments were selected from 400 base pairs upstream to 200 base pairs downstream of the transcriptional start site for each gene on condition that they had a self-complementarity score of less than 8.0 by Primer3. These probes were subjected to megaBlast and the probe nearest to the transcriptional start site, which had a score relative to the mouse genome of less than 40.0, was selected. Finally, 10,263 probes were selected. Microarrays were made using an ink-jet oligonucleotide synthesizer as described [17]. The MIAMI method was performed using 1 µg of genomic DNA as previously described [7]. The complete experimental procedure is available at http://grc.dept.med.gunma-u.ac.jp/~gene/image/MIAMI20Protocol20V4.pdf. To judge changes in methylation, we used the difference in methylation-sensitive Hpa II cleavage and methylation-insensitive Msp I cleavage between samples.

### Combined bisulfite restriction analysis (COBRA) and bisulfite sequencing

Bisulfite treatment of genomic DNA was performed using a CpGenome DNA modification kit (INTERGEN). COBRA was performed as described [18]. PCR primers and restriction enzymes are shown in Table 1. DNA fragments were separated on 8% polyacrylamide gel. PCR products were also subcloned into the TA cloning vector (pCR 2.1; Invitrogen). Ten positive clones in each sample were sequenced using the Big Dye terminator method (ABI PRISM 3100).

**Table 1:** The primer sequence used for COBRA and real-time PCR.

Site or gene name	Primer sequence	Restriction enzyme
**COBRA**		
E-box-like	5′-TTTTTTTGTATTTGATTGGTTAAAT-3′	*Hpy*CH4 IV
	5′-ATTACTAACCCAAAACCCTACCTC-3′	
*Hpa*II site1	5′-TTTTTTTGTATTTGATTGGTTAAAT-3′	*Hpy*188 I
	5′-ACCATATCAACATAACTCCAAATCC-3′	
*Hpa*II site2	5′-ATAATATTTAGGAGTTTATATTGTT-3′	*Hpy*188 I
	5′-AACCCAAAACCCTACCTCATCC-3′	
**Real-time PCR**		
β-actin	5′-GATGCAGAAGGAGATCACTGC-3′	-
	5′-GTACTTGCGCTCAGGAGGAG-3′	-
WGEF	5′-ACATCCTGGCAGTGAAGACC-3′	-
	5′-ACTCTCTGAGGTTGCGCAGT-3′	-
PPAR-γ	5′-CTGTCGGTTTCAGAAGTGCCT -3′	-
	5′-CCCAAACCTGATGGCATTGTGAGACA-3′	-
Adipsin	5′-TCCGCCCCTGAACCCTACAA-3′	-
	5′-TAATGGTGACTACCCCGTCA-3′	-
Gapdh	5′-AATGCATCCTGCACCACCAA-3′	-
	5′-GTGGCAGTGATGGCATGGAC-3′	-

### Quantitative real-time RT-PCR

Cells were collected and total RNA was extracted with TRIZOL (Invitrogen) according to the manufacturer's instructions, and RNA was treated with DNaseI at 37°C for 15 min to remove genomic DNA contamination. Total RNA was reverse transcribed using Superscript II (TaKaRa) and Oligo(dT)12–18 primer (TaKaRa) in a total volume of 20 µL. Quantitative real-time PCR was performed using SYBR Premix Ex Taq (Perfect Real Time, TaKaRa). The PCR cocktail was heated initially at 95°C for 10 sec to activate it. Subsequent PCR reaction was carried out at 95°C for 5 sec and at 60°C for 30 sec for 40 cycles in an ABI 7700 sequence detector. In each run, the dilution series of cDNA from 3T3-L1 cells were amplified to serve as a standard curve for the calculation of relative quantities of the target gene using Sequence Detector Software v1.7 (comparative CT method). All results were obtained from at least two independent experiments and each assay was duplicated. The primers used in the experiments are shown in Table 1. The mRNA levels of all genes were normalized using β-actin as an internal control.

### Luciferase Reporter Assays

The upstream region of the WGEF gene containing exon 1 (−500 to +167) was amplified by PCR from the genomic DNA of 3T3-L1 cells, and the fragment was cloned into pGL3-Basic vector (Promega). Reporter constructs with 10 nucleotides deletions around the –81 (E-box-like site), −40 and +120 (*Hpa* II site2) positions were also produced by restriction enzyme and modified PCR primer sets. 3T3-L1 cells were grown in 24-well plates and 720 ng of the luciferase reporter construct per well was transfected using Lipofectamine 2000 (Invitrogen) according to the manufacturer's directions. To normalize the luciferase activity, 80 ng of a control plasmid having a Renilla luciferase sequence was co-transfected into the cells. Cells were harvested 48 h post-transfection for 3T3-L1 cells in reporter lysis buffer (Promega). Luciferase expression was detected with a luciferase assay system (Promega).

### Plasmids and transfections

The pIRES2-EGFP mammalian expression vector (Clontech) containing the mouse WGEF cDNA sequence was generated using PCR with primers specific for ORF of the WGEF cDNA. Cells were transfected with Lipofectamine 2000 (Invitrogen) according to the manufacturer's directions. As a control, the expression vector without WGEF cDNA was also transfected. Pools of cells were stably selected with G418 (800 µg/mL) prior to experimental analysis.

### Oil red O staining

Oil Red O (ORO) staining for lipids was performed on 3T3-L1 cells that were fixed with 10% formalin in PBS for 10 min and washed in PBS. The cells were then incubated with 90 mg/mL Oil Red O (Sigma) solution dissolved in 60% 2-propanol for 20 min at room temperature.

### Statistical analysis

Data analyses were performed using Statcel2 for Macintosh OS X. All data are presented as the mean +/− standard deviations. Statistical analyses of the luciferase reporter assay and real-time PCR were performed using the Tukey-Kramer method and Student's t-test, respectively. Differences were considered significant when P<0.05.

## Results

### DNA methylation changes during the adipocyte differentiation of 3T3-L1 cells

**Figure 1:**
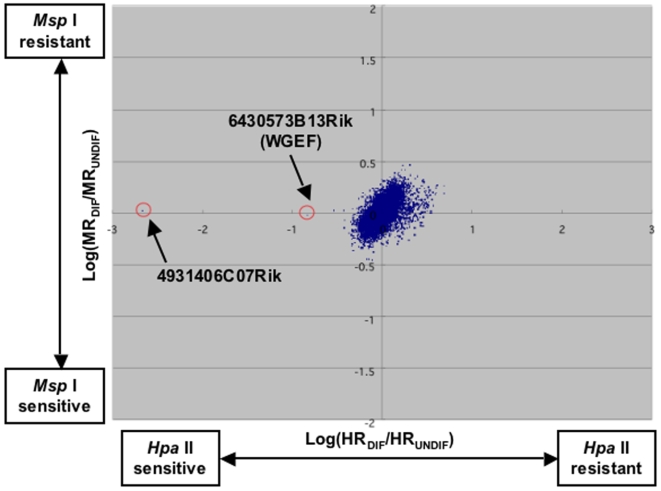
Application of the MIAMI method to 3T3-L1 cells. We applied the MIAMI method to 3T3-L1 cells that were differentiated (DIF) and undifferentiated (UNDIF). We defined resistance as reciprocal sensitivity. Therefore, *Hpa* II-sensitive (cleavable) DNA and *Msp* I-sensitive (cleavable) DNA were amplified and used to calculate *Hpa* II resistance (HR) and *Msp* I resistance (MR), respectively. Values of log (HR_DIF_/HR_UNDIF_) are plotted on the *x*-axis and log (MR_DIF_/MR_UNDIF_) are plotted on the *y*-axis. Two genes indicated by arrows are clearly hypomethylated after differentiation.

**Figure 2:**
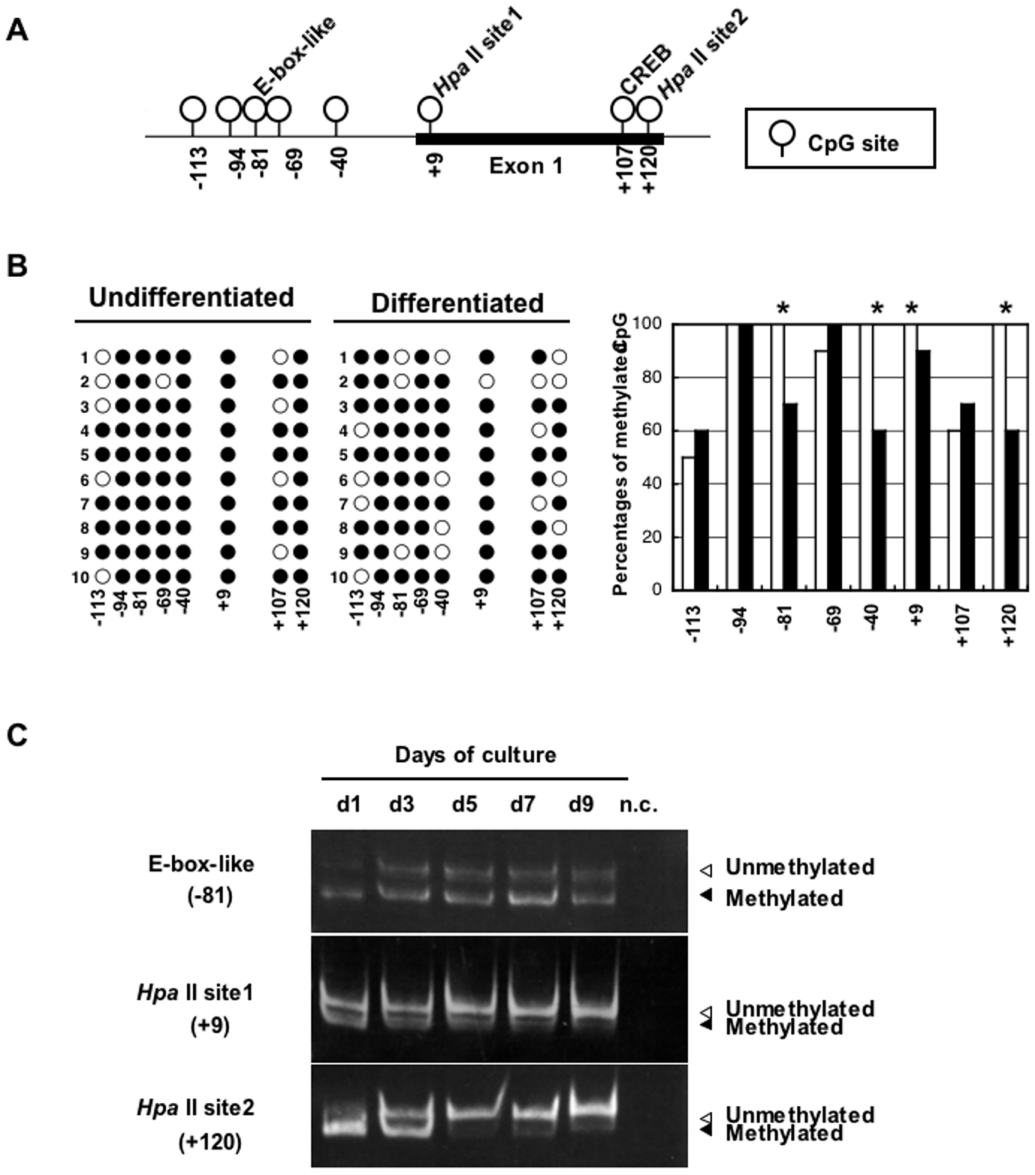
DNA methylation change of WGEF gene during adipogenesis. A. CpG sites located on exon 1 and upstream region of WGEF gene. Circles and numbers indicate positions of CpGs relative to transcription start site (+1). B. DNA methylation profiles of individual CpG sites at WGEF upstream region in undifferentiated and differentiated (day 4 of differentiation culture) 3T3-L1 cells by sodium bisulfite sequencing. Each PCR product was subcloned, and 10 clones were subjected to nucleotide sequencing analysis. The status, either unmethylated (open circle) or methylated (closed circle), is indicated at each CpG site. Methylation percentages of undifferentiated cells (open bar) and differentiated cells (closed bar) at each CpG site are summarized on the right. *CpG sites that were demethylated after differentiation. C. COBRA analysis of indicated CpG sites. PCR products with methylated CpG are digested by restriction enzymes.

Changes in methylation during adipocyte differentiation were analyzed by comparing undifferentiated and differentiated 3T3-L1 cells using the MIAMI method. The probes are located on Hpa II fragments of less than 600 base pairs and cover 10,263 genes. Hpa II cleavage differences are related to methylation differences of two samples before and after differentiation. We found that 6430573B13Rik (WGEF) and 4931406C07Rik genes clearly showed demethylation after differentiation (Fig. 1). As a result of RT-PCR, significant changes of mRNA expression during differentiation were observed in WGEF gene but not in 4931406C07Rik gene (data not shown). RhoGEF families, including WGEF, have important roles in many physiological and pathophysiological mechanisms via the activation of Rho GTPases; therefore, we performed more detailed analyses of the WGEF gene. Two Hpa II sites located on both sites of the probe are located in exon 1 of the WGEF gene (Fig. 2A). We also performed bisulfite sequencing and COBRA for the WGEF gene and found that the methylation status showed good agreement with that of MIAMI results. In particular, Hpa II site2 (120 bp downstream of exon 1 start site; +120) was almost completely methylated before differentiation, whereas strong demethylation was shown after days 5 of differentiation culture prior to full differentiation (Fig. 2B, C). Moreover, 5′-CACGTT-3′ E-box-like site [19], Hpa II site1 and −40 position also showed slight demethylation. In contrast, clear methylation changes were not seen in CREB [20] and at −69 and −94 positions.

### Expression changes during adipocyte differentiation of 3T3-L1 cells

**Figure 3:**
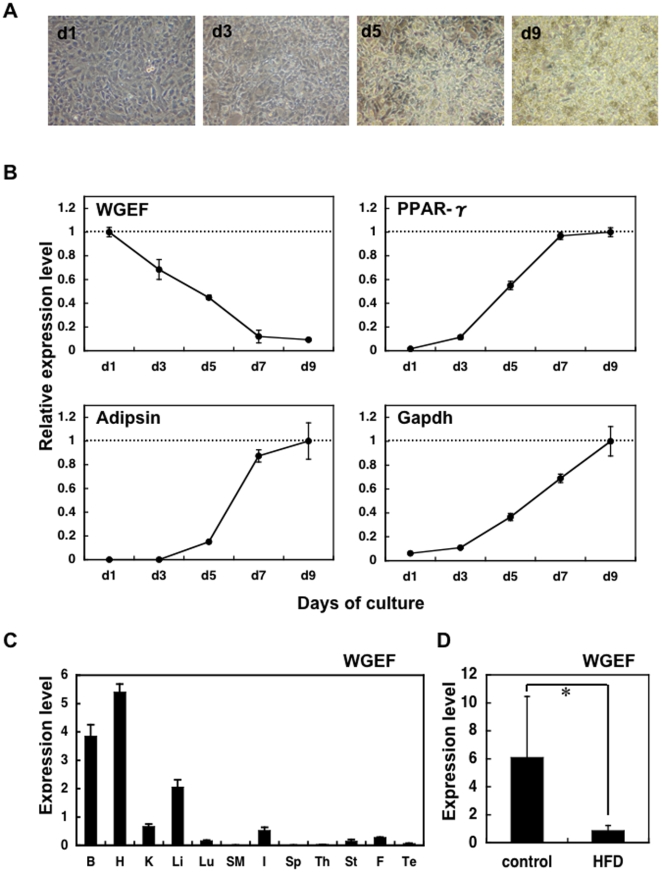
Reduction of WGEF mRNA expression during adipogenesis. A. Representative cell morphology of 3T3-L1 cells at days 1, 3, 5 and 9 of differentiation culture. Lipid droplets appeared by days 5 of culture. B. Quantitative real-time RT-PCR was performed for WGEF, PPAR-γ, Adipsin and Gapdh. Relative expression of each gene was estimated at days 1, 3, 5, 7 and 9 of differentiation culture. The β-actin gene was used to standardize the data. Data represent the mean +/− standard deviations in triplicate. C. WGEF mRNA distribution in mouse tissues. Quantitative real-time RT-PCR was performed for the brain (B), heart (H), kidney (K), lung (Lu), skeletal muscle (SM), intestine (I), spleen (Sp), thymus (Th), stomach (St), fat (F) and testis (Te). The β-actin gene was used to standardize the data. Data are the mean +/− standard deviations in triplicate. D. WGEF mRNA expression in white adipose tissue of control mice or high-fat diet (HFD)-induced obese mice (n = 6, respectively). The β-actin gene was used to standardize the data. Data are the mean +/− standard deviations in triplicate: *P<0.05

Expression changes during adipogenesis were analyzed by quantitative real-time RT-PCR. We observed over 50% reduction in WGEF mRNA at day 5 of differentiation culture when lipid droplets appeared and the methylation change was completed, and finally the relative expression level decreased to approximately 10% (Fig. 3A, B). In contrast, adipogenic markers, PPAR-γ [21], Adipsin [22] and Gapdh [23] were upregulated during adipogenesis. We also investigated the expression pattern of the WGEF gene in various mouse tissues. High expression was seen in the brain, heart and liver, and also at a lower level in the kidney and intestine (Fig. 3C). On the other hand, less expression was seen in the skeletal muscle, spleen, fat and testes. Overall, this result is identical to the result of Northern blot analysis, as reported [9]. Interestingly, lower expression of WGEF mRNA was observed in white adipose tissue of high-fat diet-induced obese mice (Fig. 3D) which have more hypertrophic adipose cells [24]. These data suggest that WGEF gene is related to inhibition of adipocyte differentiation both *in vitro* and *in vivo*.

### Regulation of transcriptional activity

**Figure 4:**
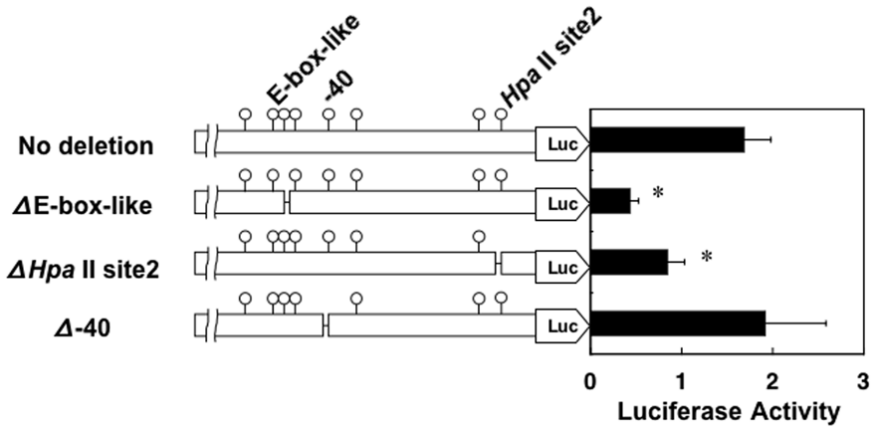
Transcriptional activity regulated by WGEF upstream region. WGEF upstream region containing exon 1 was ligated to pGL3-Basic vector. Reporter constructs with 10 nucleotides deletions (−81, −40 and +120 positions) were produced. These reporter constructs were transfected to 3T3-L1 cells. Transcriptional activity is shown as relative luciferase (Luc) activity. Data are the mean +/− standard deviations in triplicate of three independent experiments: *P<0.05.

The highest expression of WGEF mRNA was observed in undifferentiated 3T3-L1 cells, and this expression decreased during differentiation. These findings indicate the possibility that methylation change in exon 1 and the upstream region regulates transcriptional activity of the WGEF gene; therefore, luciferase reporter vectors with 10 nucleotides deletions around the −81 (E-box-like site), −40 and +120 (Hpa II site2) positions of these regions were examined for transcriptional activity (Fig. 4). Interestingly, deletions of these areas downregulated luciferase activity except for the −40 position, indicating that both −81 and +120 positions regulate mRNA expression of the WGEF gene.

### Inhibition of adipogenesis of 3T3-L1 cells by WGEF

**Figure 5:**
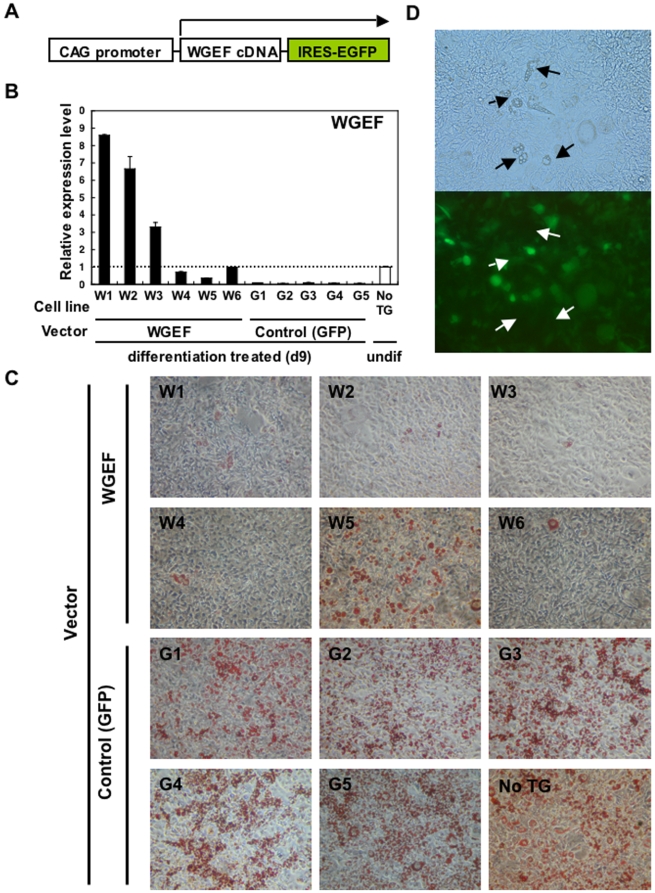
WGEF inhibits adipogenesis in 3T3-L1 cells. A. Expression vectors with/without WGEF cDNA were individually transfected into 3T3-L1 cells, which were subsequently selected in G418-containing medium. Then, GFP-positive clones were analyzed. B. Quantitative real-time RT-PCR was performed for total (endogenous and transgenic) WGEF. Relative expression of each cell line was estimated at day 9 of differentiation culture. The β-actin gene was used to standardize the data. Data are the mean +/− standard deviations in triplicate. C. Oil Red O staining of WGEF- and vector-transfected 3T3-L1 cells subjected to 9 days of differentiation. Vector control cells exhibited significantly more stained lipid droplets than WGEF-transfected cells, except for the W8 cell line which showed less than 50% WGEF expression compared to undifferentiated No TG cells. D. Cells with high expression of WGEF (GFP) were not differentiated, whereas those with a low expression of WGEF (GFP) differentiated and produced lipid granules (arrows) even in the same cell line.

**Figure 6:**
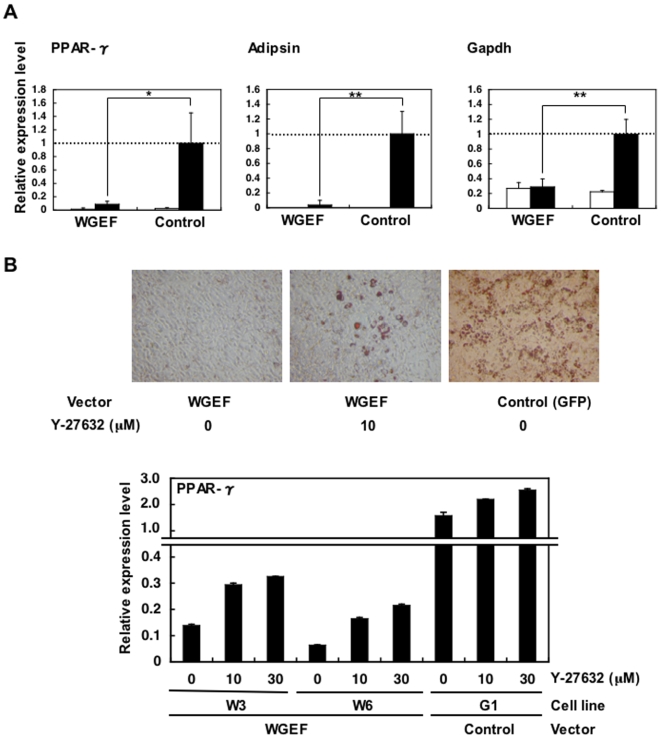
WGEF inhibits adipogenesis through the RhoA-ROCK signaling pathway. A. 3T3-L1 cells were stably transfected with WGEF or control vector (GFP). Quantitative real-time RT-PCR was performed for adipogenic markers PPAR-γ, Adipsin and Gapdh. Relative mRNA expression levels before (open bars) and after (closed bars) differentiation treatment are shown. Data are the means +/− standard deviations (n = 5, respectively): *P<0.005 and **P<0.0005. B. WGEF-transfected cells (W3 and W6) were treated with the ROCK inhibitor, Y-27632. Oil Red O staining (Top) and quantitative real-time RT-PCR for PPAR-γ (bottom) show that adipogenesis was induced after treatment with Y-27632 in a dose-dependent manner. Data are the means +/− standard deviations.

Our data demonstrate strong down-regulation of WGEF expression during 3T3-L1 adipogenesis, suggesting that this gene may be essential for maintaining the undifferentiated state of these cells. In order to determine if WGEF can regulate the process of adipogenesis, we made six WGEF-transfected stable cell lines (W1–W6) and five vector control (GFP-transfected) stable cell lines (G1–G5) (Fig. 5A, B). These WGEF-transfected cell lines expressed WGEF even after 9 days of treatment for differentiation (Fig. 5B). As a visual measure of the effect of WGEF on adipogenesis, we carried out Oil Red O (ORO) staining to detect lipid granules in 3T3-L1 cells. Fully differentiated 3T3-L1 cells produce lipid granules that can be stained with ORO. We found that vector control cells exhibited clearly more stained lipid droplets than WGEF-transfected cells (Fig. 5C). According to real-time RT-PCR analysis, excessive expression of WGEF inhibited adipogenesis, whereas over 50% reduction in WGEF expression could not inhibit (i.e. W5 cell line). Further, in the same stable cell line, cells with a high expression of WGEF were not differentiated, whereas those with a low expression produced lipid granules (Fig. 5D). Additionally, real-time RT-PCR analysis of the adipogenic markers PPAR-γ, Adipsin, and Gapdh demonstrated strong inhibition of gene expressions in WGEF-transfected cells compared to vector-transfected cells (Fig. 6A), demonstrating that WGEF is capable of inhibiting the adipogenic program. Generally, activated RhoA effect downstream target, ROCK which inhibits adipogenesis by control of cytoskeletal tension [16]. If overexpression of WGEF inhibited adipogenesis through the RhoA-ROCK signaling pathway, adipogenesis could be induced by inhibition of ROCK in WGEF-transfected cells. We treated WGEF transfected cells with Y-27632 which was known as a ROCK inhibitor, and we found that adipogenesis was induced in a dose-dependent manner with concentration of 0 µM, 10 µM and 30 µM. This indicates that the WGEF-RhoA-ROCK signaling pathway could inhibit adipogenesis in 3T3-L1 cells.

## Discussion

Epigenetic regulation, including DNA methylation, plays an important role in several differentiation processes. We profiled global DNA methylation during adipocyte differentiation of 3T3-L1 cells using a microarray-based method called MIAMI, and found dramatic methylation change in the WGEF gene. Detailed analyses clarified that methylation changes of WGEF were completed prior to complete repression of the expression during adipocyte differentiation. This finding indicates the possibility that methylation change in exon 1 and the upstream region regulates transcriptional activity of the WGEF gene. The results of luciferase reporter assays show evidence that the region containing −81 (E-box-like site) and +120 (*Hpa* II site2) positions regulates WGEF transcription.

Generally, chromatin modification by CpG methylation has been linked with transcriptional repression [25]. In contrast, transcription of WGEF has been upregulated by CpG methylation. At first glance, the relationship between methylation and transcription in WGEF seems to be contradictory; however, there are some reports that methylation in the 5′ or 3′ region activates gene expression. In human osteoblast-like MG63 cells, for example, methylation in the distal promoter part of the PDPN gene promotes PDPN transcription efficiently [26]. They hypothesized that methylation-dependent protein-DNA interactions stimulate transcription. The zinc finger protein Kaiso has also been found to bind specifically to methylated sites in a sequence-specific manner, activating functions [27]. Thus, the existence of methylation-dependent proteins that promote transcription was reported by some researchers. In the case of WGEF, methylation-dependent binding proteins able to promote transcription may bind to the upstream region of the WGEF gene. In fact, deletion of the region containing E-box-like site and *Hpa* II site2 suppressed transcriptional activity in the reporter assay, indicating that this region regulates WGEF transcription at least.

In expression analysis, our data demonstrate a strong down-regulation of WGEF expression during 3T3-L1 adipogenesis, suggesting that this gene may be essential for maintaining the undifferentiated status of these cells. High-fat diet-induced obese mice showed lower expression of WGEF than control mice in white adipose tissue, supporting this idea. According to expectations, an excessive or equivalent level forced the expression of WGEF compared to undifferentiated cells that inhibited adipocyte differentiation; however, more than 50% reduction of expression level led to differentiation. Additionally, using real-time RT-PCR analysis measuring adipogenic markers PPAR-γ, Adipsin and Gapdh, we observed a strong inhibition of marker gene expression in WGEF-transfected cells compared to vector control transfected cells. Thus, we have obtained the first evidence that WGEF signaling pathways act as potential regulators of adipogenesis.

WGEF can activate Rho family G-proteins such as RhoA, Rac1 and Cdc42 [9]. Another study shows that RhoA inhibits adipogenesis by activation of the RhoA effector, ROCK which regulates cytoskeletal tension by actin polymerization [16]. These two studies indirectly suggest the inhibition of adipogenesis by WGEF-RhoA-ROCK signaling. In our study, WGEF-transfected cells did not start adipogenesis, whereas they started adipogenesis after treatment with RhoA-ROCK inhibitor, Y-27632. This result directly shows that the inhibition of adipogenesis is caused by WGEF-RhoA-ROCK signaling. As noted above, RhoGEFs, including WGEF, activate Rho GTPases. In contrast, RhoGAPs stimulate GTP hydrolysis, which results in the inactivation of Rho GTPases. Interestingly, mice lacking p190-B RhoGAP show a dramatic reduction of mature adipocytes because excessive Rho activity in the absence of RhoGAPs inhibits adipogenesis [28]. In other words, their report suggests that excessive RhoGEF expression inhibits adipogenesis. Furthermore, it has been reported that another member of RhoGEFs, GEFT, also inhibits adipogenesis in 3T3-L1 cells via the activation of RhoA [29]. According to their report, cotransfection of GEFT with the PPAR-γ luciferase construct resulted in a reduction from baseline luciferase activity. Our results also indicated the strong inhibition of PPAR-γ transcription by the forced expression of WGEF (Fig. 6A). WGEF transfected cells that were treated with the ROCK inhibitor, Y-27632 certainly started adipogenesis; however, the differentiation level of these cells did not reach that of normal control cells (Fig. 6B). This suggests that WGEF blocks the adipogenic program via not only the RhoA-ROCK signaling pathway but also via other pathways, such as the PPAR-γ-mediated signaling pathway which is one of the most critical programs of adipogenesis. In any case, it is clear that WGEF plays a very important role in adipogenesis through the WGEF-RhoA-ROCK signaling pathway by epigenetic regulation.
